# Prevalence and Risk Factors of Depression, Anxiety, and Stress in a Cohort of Australian Nurses

**DOI:** 10.3390/ijerph16010061

**Published:** 2018-12-27

**Authors:** Shamona Maharaj, Ty Lees, Sara Lal

**Affiliations:** Neuroscience Research Unit, School of Life Sciences, University of Technology Sydney, 15 Broadway, Ultimo, Sydney, NSW 2007, Australia; shamona.maharaj@uts.edu.au (S.M.); ty.lees@uts.edu.au (T.L.)

**Keywords:** mental health, nursing, depression, anxiety, stress

## Abstract

Nurses remain at the forefront of patient care. However, their heavy workload as a career can leave them overworked and stressed. The demanding nature of the occupation exposes nurses to a higher risk of developing negative mental states such as depression, anxiety, and stress. Hence, the current study aimed to assess the prevalence and risk factors of these mental states in a representative sample of Australian nurses. The Depression Anxiety Stress Scale was administered to 102 nurses. Information about demographic and work characteristics were obtained using lifestyle and in-house designed questionnaires. Prevalence rates of depression, anxiety, and stress were found to be 32.4%, 41.2%, and 41.2% respectively. Binominal logistic regressions for depression and stress were significant (*p* = 0.007, *p* = 0.009). Job dissatisfaction significantly predicted a higher risk of nurses developing symptoms of depression and stress respectively (*p* = 0.009, *p* = 0.011). Poor mental health among nurses may not only be detrimental to the individual but may also hinder professional performance and in turn, the quality of patient care provided. Further research in the area is required to identify support strategies and interventions that may improve the health and wellbeing of nursing professionals and hence the quality of care delivered.

## 1. Introduction

The health workforce in Australia is dominated by nurses, making them indispensable to the healthcare sector [1,2]. Nurses not only provide care and assistance to patients, but also participate in their rehabilitation, provide support to patients and their families, and advocate health education [3], playing an important role in improving and promoting health services in the community [4]. Their broad and multifaceted workload presents them with the largest amount of time spent with patients and allows them to cover all areas of the healthcare network [3]. Yet, the profession is invariably recognised as a stressful and demanding occupation.

Nurses regularly experience a variety of work-related stressors including but not limited to: long work hours, time constraints, meeting patients’ needs, irregular schedules, and lack of professional support [2,5,6,7]. With such demanding occupations, the ongoing strain faced by healthcare professionals could have a severe impact on their mental health and quality of life [8,9]. Previous studies have shown that the ongoing stress faced by these professionals can have negative effects on their psychological well-being [10,11]. Poor mental health among health care providers may also hinder their professional performance and have a considerable effect on the quality of care they provide to patients [2,10] (inevitably impacting negatively on patients’ health). Therefore, administrators and nursing managers are becoming increasingly interested in the health status of nurses.

Psychological indicators for distress include but are not limited to, low self-esteem, fatigue, and sleep/appetite disturbances [12]; while physical impacts may include the increased risk of cardiovascular disease, high blood pressure, decreased immunity, migraines, muscle aches, and chronic fatigue [13]. Additionally, high levels of stress may lead to or exacerbate maladaptive behaviours, such as smoking, over/under eating, excessive alcohol consumption, and substance abuse [14,15]. Depression and anxiety also remain two of the most prevalent mental disorders in Australia with 12-month prevalence rates of approximately 4% and 14% respectively [16]. Investigating depression, anxiety, and stress levels in nurses and identifying predictors for these mental states is crucial for future health systems to provide safer and more amenable workplaces while promoting the well-being of its employees.

The prevalence of depressive symptoms among nurses in the USA was between 35–41%, but another study reported it to be 18% [7,10], 11%–80% in Iranian nurses [17,18], 35% in Chinese nurses [19], 17% in Australian Midwives [20], and 51% in Brazilian nurses [21]. Additionally, approximately 33% of French nurse managers and 10% Canadian nurses were found to suffer from depressive symptoms as well [22,23]. A high incidence of anxiety in nursing professionals is also evident, with studies stating prevalence rates ranging from 20% in Australian Midwives [20] to 32–43% in Chinese nurses [11,19,24], 40–46% in Iranian nurses [4,25], 44–66% in Brazilian nurses [21,26], and 22–24% of American nurses who showed PTSD symptomology [27,28]. The level of stress among nurses can generally range from moderate to high [29,30] and the few prevalence rates that have been presented range from approximately 40–90% [31,32,33,34]. Predictive factors such as job satisfaction, high workloads, shift work, sleep disturbance, years of employment, and marital status [10,17,18,19,35] were commonly implicated with these mental disorders.

Hence, the current study aimed to

1. Assess the prevalence of depression, anxiety, and stress in a cohort of Australian nurses.
2. Determine demographic and work characteristics associated with each mental state.

## 2. Materials and Methods

### 2.1. Participants and Sampling

A cross-sectional study design was used to examine the prevalence and correlates of depression, anxiety, and stress symptoms in a cohort of Australian nurses. Data from 102 clinically active nurses were used in the current analysis. Prior to inclusion, all participants were screened for chronic disease/illness (including clinically diagnosed depression, anxiety, and other mental health issues), medication use, smoking habits (in excess of 10 cigarettes a day), and alcohol intake (in excess of 16 standard drinks a day) via an in-house designed lifestyle questionnaire. Participants were excluded if they answered yes to any of the screening questions above. All participants provided informed consent prior to the commencement of the study. At the commencement and conclusion of each session, the participant’s blood pressure (BP) was measured three times with an automated blood pressure monitor (Omron IA1B, Kyoto, Japan), and then averaged to derive an average BP value to determine further inclusion into the study (requiring BP < 160/100 mmHg).

### 2.2. Data Collection/Measures

Demographic, lifestyle, and work-related data were collected using in-house designed questionnaires; developed to determine lifestyle risk factors/habits and determine inclusion/exclusion criteria for the study.

The 42-item Depression Anxiety Stress Scale (DASS) [36] was applied to assess symptom severity of each mental state. Together, each scale of the DASS assessed a comprehensive range of psychological distress symptoms to identifying psychological disturbances perceived by an individual ranging from situational anxiety, anhedonia, and dysphoria to levels of chronic non-specific arousal such as irritability and difficulty relaxing [36,37]. It can be noted that perceived job strain and control are commonly associated with self-reported mental health outcomes [38,39]. As the scale examines general mental health and is based on self-perception, it accounts for environmental and work-related factors that may influence how an individual is feeling.

### 2.3. Statistical Analysis

Data was initially subject to descriptive statistical analysis. Pearson’s correlation analysis was conducted to assess associations between blood pressure and Depression, Anxiety, and Stress. Variables were then dichotomised to enable a good comparison of outcomes; participants with a cut-off score of ≥10 in depression, ≥8 in anxiety, and >15 in stress were considered as having these disorders as referenced by the DASS [36]. A forward-stepwise (conditional) binominal logistic regression was utilised to measure the strength of associations between variables and sought to identify significant predictors for the outcomes of interest to the study. The level of statistical significance was defined as *p* < 0.05. Statistical analysis was performed using SPSS Version 23.0 for the Windows platform (SPSS Inc.; Chicago, IL, USA).

## 3. Results

### 3.1. Demographics

Participants demographics are presented in Table 1. As seen in Table 1, a majority of the participants were females working as Assistants in Nursing (AIN’s) and Registered nurses. A majority of the participants also worked in aged care and hospital environments, reflecting the large number of AIN’s and registered nurses. Finally, most of the nurses assessed in the current study engaged in shiftwork.

Mean DASS scores and prevalence for each mental state are displayed in Table 2. In the total sample, according to DASS scores, 32.4% of participants scored over the normal threshold for depression. Of those individuals, 21.57% fell into the mild/moderate categories while 4.9% showed sufficient scores for severe depression and 5.88% for extremely severe depression. However, though some individuals did not have scores over the normal threshold, 86.27% of the total cohort did report some level of depressive symptoms.

Similarly, 41.2% of participants scored over the normal threshold for anxiety. Of those individuals, 20.59% fell into the mild/moderate categories while 9.8% showed sufficient scores for severe anxiety and 10.78% for extremely severe anxiety. Though not all the scores were over the threshold, 91.18% of the total cohort did report some level of anxiety.

Finally, 41.2% of participants also scored over the normal threshold for stress. Of those individuals, 24.51% fell into the mild/moderate categories while 10.8% showed sufficient scores for severe stress and 5.88% for extremely severe stress. Once again, though some individuals did not score over the threshold, 95.10% of the total cohort did report some level of distress.

As previously mentioned, blood pressure was assessed for inclusion criteria. Associations between blood pressure and depression, anxiety, and stress were non-significant, as seen in Table 3.

### 3.2. Binary Logistic Regression Models

The logistic regression model for depression was significant (*p* = 0.007), explaining 14.2% (Nagelkerke R2) of the variance in depression, and correctly classified 67.7% of cases. Job dissatisfaction significantly predicted depression (S.E. = 0.538, Exp(B) = 4.07, df = 1, *p* = 0.009) with individuals who reported not being satisfied being more likely to present with depression.

The logistic regression model for anxiety was non-significant. The association between the study variables showed no significance between anxiety and the following: age, gender, facility, shift work, shift type, shift length, job satisfaction, physical issues, and registration level.

The logistic regression model for stress was significant (*p* = 0.009), explaining 13.2% (Nagelkerke R2) of the variance in stress, and correctly classified 66.2% of cases. Job dissatisfaction significantly predicted stress (S.E. = 0.53, Exp(B) = 3.85, df = 1, *p* = 0.011) with individuals who reported not being satisfied also being more likely to present with symptoms of distress.

## 4. Discussion

We assessed the status of depression, anxiety and stress symptomology in a representative sample of Australian nurses. Our study demonstrated that depressive symptoms were common in nurses with a prevalence rate of over 30%, compared to only 4% of the general Australian population [16]. Thus, the results suggested that more than a quarter of the current sample complied with DASS cut-off criteria for depression (scores of 10 and above). Notably, multiple had presented with severe depressive symptoms. Depression prevalence among nurses in our study was also within the ranges reported in previous literature around the world with depression rates ranging from approximately 18–53% [10,18,19,20,21]. Likewise, the prevalence of anxiety symptoms was common among the current sample, with a prevalence rate of over 40% compared to 14% of the general Australian population [16]. Again, more than a quarter of the current sample complied with the DASS cut off criteria for anxiety (8 and above) and among these individuals, over 10% had presented with severe anxiety. The prevalence of anxiety among nurses in our study also fell within the ranges commonly reported in the literature (ranging between 20–60%) [4,11,19,20,21,24,26,29]. Finally, levels of distress were high with over 40% of the current nursing sample complying with the DASS cut-off criteria for stress (scores of 15 and above) and once again multiple individuals presented with severe levels of distress while our prevalence rate was similar to those of other countries [32,34].

As previously mentioned, rates of depression, anxiety, and stress in the current cohort were much higher than population norms. Ignoring the signs of anguish and depression presented by nursing professionals may not only increase the amount of physical and emotional stress on the individual but may also result in low-quality patient care and higher work burdens on establishments [40]. Literature suggests that poor mental health may lead to a decrease in cognitive performance such as an individual’s ability to focus and process information, in turn, resulting in inadequate performance [41,42]. Such consequences in the workplace can include decreased alertness and reduced job performance, which could endanger human lives and increase the risk of adverse medical events [43,44].

Australian state spending on mental health services has increased over the past few years, now costing approximately $9 billion [45] and more notably, the cost and number of workers’ compensation claims associated with stress and stress-related mental disorders are noticeably higher in medicine and healthcare compared to other occupations [46,47]. Individuals who suffer from mental health issues also tend to have more days out of role, taking approximately five days off work and having 11 days of reduced productivity annually; adding billions of dollars lost to absenteeism and lost productivity [48,49]. Although the number of nurses in Australia has increased over the last decade [50], nurse shortages remain an ongoing issue [51]. High turnover, absenteeism, and lost productivity within the health workforce could amplify shortages and easily leave facilities understaffed and unable to meet patient demands, placing patients at risk [3,52]. Thus, as the prevalence of mental health disorders increases within the profession so too will the economic, social, and individual impacts of these disorders.

The current findings suggested that demographic and occupational factors associated with an increased incidence of developing symptoms of depression, anxiety, and stress were limited as only one occupational factor was associated with poor mental health outcomes. While job dissatisfaction was associated with an increased risk of distress and depression, no factors were found to significantly increase the likelihood of developing anxiety. Further, blood pressure parameters were similarly not associated with depression, anxiety, or stress. However, previous literature has implicated various demographic and work-related predictors for the relatively high prevalence of negative mental states among nurses. Previous studies have found common predictors such as age, job satisfaction, sleep disturbance, years of employment, and marital status [10,17,18,19,53] to increase the likelihood of developing stress, anxiety and depression. Associations between highly stressful work and its impact on mental wellbeing necessitate the consideration of interventions which aim to improve working conditions and reduce the personal and occupational stress of nurses in an aid to reduce and/or prevent symptoms of depression, stress, and anxiety [43].

Limitations (such as a small representative sample and cross-sectional study design) do exist in the current study, and hence further research may benefit from longitudinal study designs to fully determine predictors of stress, depression, and anxiety in nurses. As the current data was collected as part of a larger study, limited information was collected and available for analysis. A more comprehensive assessment of demographic, personal, and work-related factors that may be considered predictors for negative mental states may also be beneficial.

## 5. Conclusions

Nursing is an important role in our health care system, and patient care is heavily reliant upon their ability to work optimally and deliver the best care possible [54]. It is becoming increasingly recognised that nurses are affected by symptoms of stress, depression, and anxiety [9]. The prevalence of nurses affected by negative mental states in the current study was high, and it is possible that poor mental health can be detrimental to both the individual and the industry [2,10]. Further research is needed to help care for the well-being of nurses and minimize poor mental health in the workplace. Additionally, the development of short and long-term support strategies and interventions aimed at improving the mental health needs of nursing professionals should be a priority to combat the physical and psychological exhaustion associated with these mental states.

The stress, depression, and anxiety experienced by nursing professionals may not be entirely preventable but realising its prevalence in the workplace is considerably important [55]. A healthy workforce is essential in ensuring that both personal wellbeing and quality patient outcomes are achieved [55].

## Figures and Tables

**Table 1:** Demographics (n = 102).

Demographic/work factor	Mean ± SD or %
Age	30.91 ± 11.52
Gender (female)	79.41% (81F: 21M)
Position	
Assistant in Nursing	43.14% (44)
Enrolled nurse	15.69% (16)
Registered Nurse/Midwife	39.22% (40)
Other	1.96% (2)
Facility	
Aged care	33.33% (34)
Hospital	34.31% (35)
Multiple facilities	9.80% (10)
Other	5.88% (6)
Unspecified	16.67% (17)
Shift workers	78.43% (80)
Job satisfaction	
Yes	37.25% (38)
No	26.47% (27)
Unspecified	36.27 (37)

**Table 2:** Prevalence and Average scores for Depression, Anxiety, and Stress (n = 102).

	Normal Threshold	Cohort Mean ± SD	Interpretation	Prevalence (%)
Depression	<10	8.38 ± 8.81	Normal	32.4
Anxiety	<8	8.47 ± 7.86	Mild	41.2
Stress	<15	13.90 ± 9.94	Normal	41.2

**Table 3:** Correlations between blood pressure and depression, anxiety, and stress.

	Pre Systolic	Pre Diastolic	Pre Heart Rate	Post Systolic	Post Diastolic	Post Heart Rate
Depression	r = 0.03, *p* = 0.849	r = 0.10, *p* = 0.493	r = −0.14, *p* = 0.346	r = 0.05, *p* = 0.758	r = −0.10, *p* = 0.540	r = −0.15, *p* = 0.330
Anxiety	r = 0.03, *p* = 0.848	r = 0.07, *p* = 0.646	r =−0.07, *p* = 0.632	r = 0.10, *p* = 0.492	r = −0.07, *p* = 0.647	r = 0.01, *p* = 0.931
Stress	r = 0.01, *p* = 0.929	r = 0.09, *p* = 0.537	r = 0.01, *p* = 0.959	r = 0.09, *p* = 0.554	r = −0.05, *p* = 0.728	r = −0.004, *p* = 0.979
